# Irreversible Diabetic Striatopathy in Reversible Diabetes

**DOI:** 10.1210/jcemcr/luaf292

**Published:** 2026-01-06

**Authors:** Subhankar Chatterjee, Alak Pandit, Samya Sengupta, Shambaditya Das, Ritwik Ghosh, Souvik Dubey

**Affiliations:** Department of Endocrinology & Metabolism, Medical College and Hospital, Kolkata 700073, India; Department of Neuromedicine, Bangur Institute of Neurosciences, IPGMER and SSKM Hospital, Kolkata 700020, India; Department of Neuromedicine, Bangur Institute of Neurosciences, IPGMER and SSKM Hospital, Kolkata 700020, India; Department of Neuromedicine, Bangur Institute of Neurosciences, IPGMER and SSKM Hospital, Kolkata 700020, India; Department of General Medicine, Burdwan Medical College and Hospital, Burdwan 713104, India; Department of Neuromedicine, Bangur Institute of Neurosciences, IPGMER and SSKM Hospital, Kolkata 700020, India

**Keywords:** diabetic striatopathy, steroid-induced diabetes, hemichorea, chronic kidney disease, reversible diabetes, basal ganglia

## Abstract

Diabetic striatopathy (DS) is a rare neurometabolic complication of poorly controlled diabetes, typically presenting as hemichorea-hemiballismus. It is most often associated with long-standing diabetes but can rarely occur in steroid-induced diabetes. We report the case of a 59-year-old woman with chronic kidney disease and hypertension who developed new-onset diabetes mellitus and right-sided hemichorea following corticosteroid therapy for nonproliferative glomerulopathy. Magnetic resonance imaging (MRI) confirmed DS with hyperintensity in the left lentiform nucleus. Despite initially requiring insulin, her diabetes resolved entirely following glucocorticoid withdrawal. Choreiform movements improved significantly with glycemic control and neuroleptics; however, residual hemichorea persisted. Follow-up MRI revealed gliotic changes in the striatum. This case highlights the importance of recognizing DS as a possible complication of steroid-induced diabetes. Timely identification and glycemic control can reverse both metabolic and neurological complications, although residual deficits may persist.

## Introduction

Diabetic striatopathy (DS) is a rare neurological manifestation of uncontrolled diabetes mellitus, typically presenting with hemichorea or hemiballismus [1, 2]. Although most commonly seen in elderly individuals with long-standing type 2 diabetes, acute-onset involuntary movement disorder has also been reported in other types of diabetes, including type 1 diabetes [3] and type 3c diabetes [4]. Only 3 cases of DS in the context of “secondary diabetes” have been reported in the background of an underlying endocrinological pathologies [5-7]. One was acromegaly [5], and the other 2 were glucocorticoid-induced hyperglycemia [6, 7]. Here we describe a unique case of DS occurring in the context of glucocorticoid-induced diabetes in a patient with preexisting chronic kidney disease (CKD). The case reiterates the importance of the “double-hit hypothesis” in the pathophysiology of DS (ie, acute metabolic insult on top of preexisting striatal microangiopathy).

## Case Presentation

A 59-year-old woman presented to the neurology outpatient department with a 2-month history of involuntary movements affecting the right side of her body (lower > upper). These movements began 1 month after the onset of hyperglycemia.

She had a known history of hypertension and CKD, diagnosed 4 years prior. Over this period, her renal function had ranged between stage G3a and G3b, with persistent grade A3 proteinuria. The patient never received dialysis. Her regular medications included telmisartan (40 mg/day), cilnidipine (5 mg/day), atorvastatin (20 mg/day), iron and folic acid supplements, calcium, and vitamin D3. Three months prior to presentation to the neurology clinic, she experienced worsening proteinuria [spot urinary albumin creatinine ratio (UACR) 3780 mg/gm (427.14 mg/mmol) (normal reference range: <30 mg/gm; <3.4 mg/mmol)] and a decline in estimated glomerular filtration rate (eGFR) to 40 mL/min/1.73 m² (normal reference range: ≥90 mL/min/1.73 m²). An antinuclear antibody profile test was negative. A renal biopsy revealed nonproliferative glomerulopathy, and she was started on prednisolone at 1 mg/kg/day under nephrology supervision.

Two weeks after starting corticosteroids, she developed hyperglycemia with a fasting plasma glucose (FPG) of 228 mg/dL (12.67 mmol/L) (normal reference range: <100 mg/dL; <5.56 mmol/L), postprandial glucose (PPPG) of 309 mg/dL (17.17 mmol/L) (normal reference range: <140 mg/dL; 7.78 mmol/L), and glycated hemoglobin (HbA1c) of 5.9% (41 mmol/mol) (normal reference range: <5.7%; 36 mmol/mol). Medical records confirmed that her prior glycemic values had consistently been within normal limits (FPG 92 mg/dL, 5.11 mmol/L; PPPG 129 mg/dL, 7.17 mmol/L; HbA1c 5.4%, 36 mmol/mol). Owing to new-onset hyperglycemia, her nephrologist initially started oral antidiabetic drugs, followed by twice-daily premixed insulin. However, her glycemic status remained poorly controlled.

## Diagnostic Assessment

**Figure 1.**
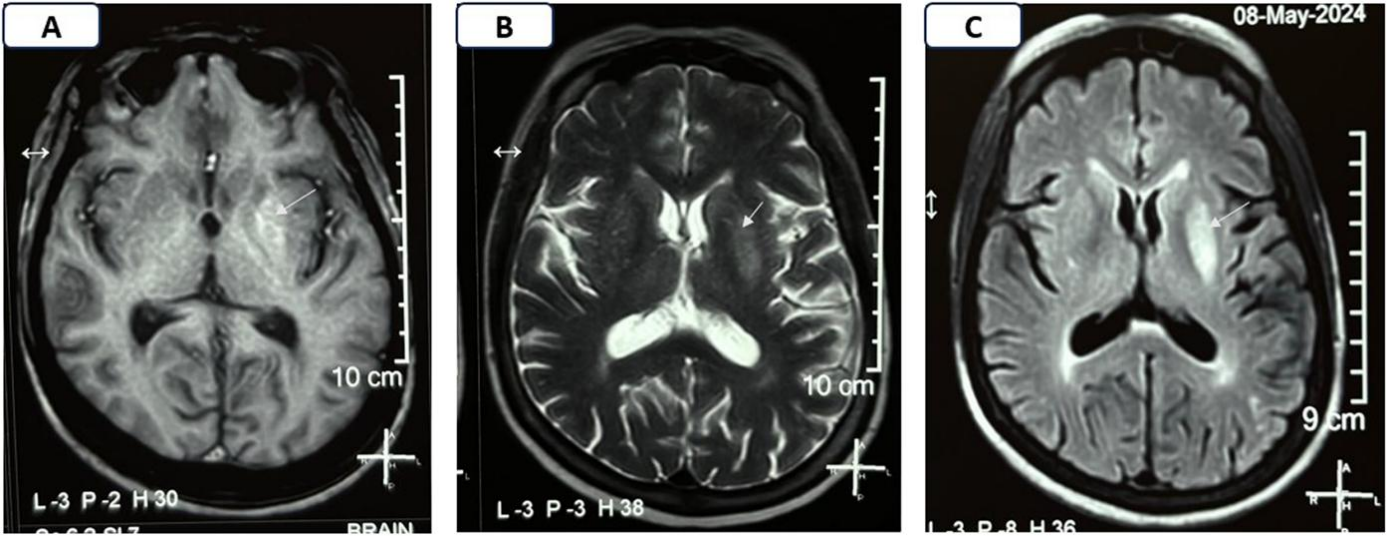
Magnetic resonance imaging of the brain done at presentation showing hyperintensity on T1, characteristic of diabetic striatopathy (A); hyperintensity on T2 (B); and fluid-attenuated inversion recovery (C) sequences in the left lentiform nucleus (white arrows).

Approximately 1 month after starting prednisolone, she developed subacute, progressive involuntary movements of the right lower limb, which gradually involved the right upper limb too. At the time of neurological consultation, she was receiving 50 mg/day of prednisolone. Neurological examination revealed brief, nonrepetitive, nonrhythmic, semipurposeful flowing involuntary movements of the right upper and lower limbs, suggestive of hemichorea. There were no other focal neurological deficits. Other systemic examination including fundoscopy was unremarkable. Laboratory investigations showed the following: random blood glucose 523 mg/dL (29.06 mmol/L) with an HbA1c of 8.5% (69 mmol/L), hemoglobin 9.9 g/dL (99 g/L) (normal reference range: 12.1-15.1 g/dL; 121-151 g/L), peripheral blood smear suggestive of normocytic normochromic anemia, serum creatinine 1.24 mg/dL (0.11 mmol/L), eGFR 50 mL/min/1.73 m², and UACR 580 mg/gm (65.54 mg/mmol). Serum electrolytes (sodium, potassium, calcium, phosphorus), liver and thyroid function tests, serum 25(OH)-vitamin D, intact PTH, ceruloplasmin, thiamine, ferrokinetics, and arterial blood gas analysis were within normal limits. Abdominal ultrasound was normal except for mild hepatomegaly, grade 1 fatty liver, and increased cortical echogenicity of normal-sized kidneys with partial loss corticomedullary differentiation. Electrocardiograph and 2D echocardiograph were unremarkable. Magnetic resonance imaging (MRI) of the brain revealed T1 hyperintensity in the left lentiform nucleus without diffusion restriction or contrast enhancement, consistent with DS (Fig. 1).

## Treatment

The patient was transitioned to a basal-bolus insulin regimen along with empagliflozin (25 mg/day) and linagliptin (5 mg/day). Tetrabenazine (25 mg twice daily) and clonazepam (0.25 mg once daily) were prescribed for symptomatic relief from chorea. With intensive glycemic control, there was marked improvement in involuntary movements within days. Prednisolone was gradually tapered over 6 weeks and eventually discontinued. As insulin and oral antidiabetic drug requirements declined, she was eventually weaned off all antidiabetic therapy. Neuroleptic medications were stopped by the patient after her symptoms became negligible.

## Outcome and Follow-up

**Figure 2.**
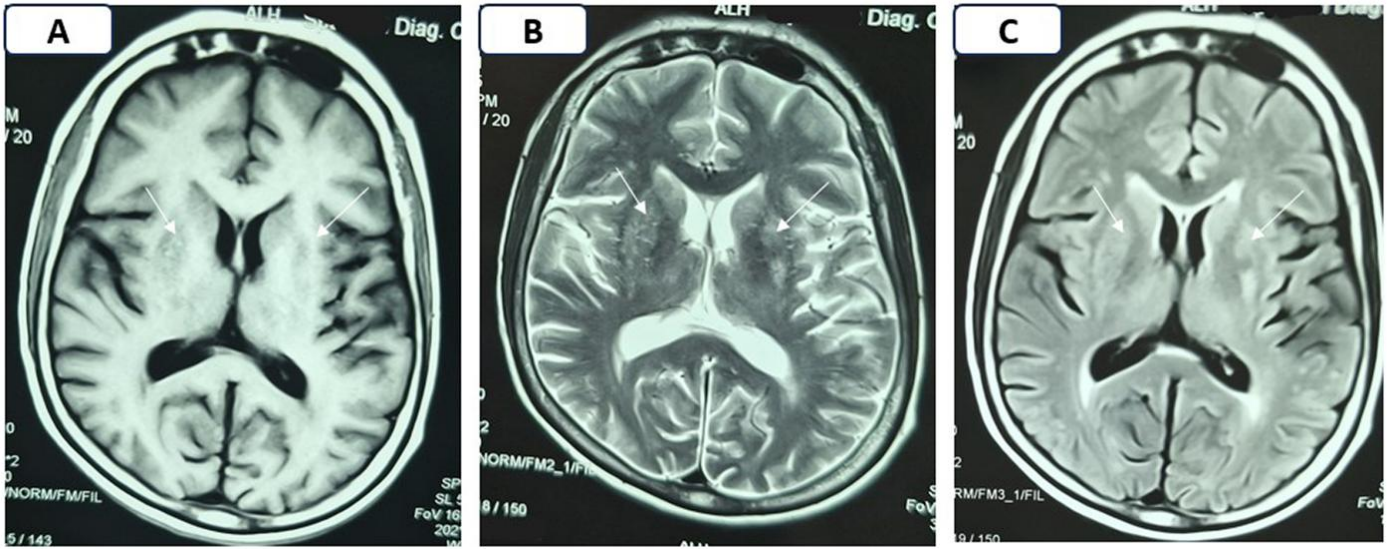
Magnetic resonance imaging of the brain done after 18 months of the first presentation showing multiple focal areas of isointense signals in bilateral ganglio-capsular regions on T1 (A) and T2 (B) weighted images with mixed signals on fluid-attenuated inversion recovery sequence (C) (white arrows), without any restriction on diffusion weighted imaging (not shown), suggestive of gliotic areas.

Over the next 18 months, she maintained normal glycemic parameters without medication [FPG 76 mg/dL (4.22 mmol/L), PPPG 98 mg/dL (5.44 mmol/L), HbA1c 5.5% (37 mmol/mol)]. Her latest serum creatinine, eGFR, and UACR were 1.33 mg/dL (0.12 mmol/L), 46 mL/min/1.73 m², and 433 mg/gm (48.93 mg/mmol), respectively. Despite complete resolution of diabetes, mild residual choreiform movements at the distal most part of the right extremity persisted, though they did not interfere with daily activities. Follow-up brain MRI revealed gliotic changes in the left striatum, indicative of permanent structural damage (Fig. 2). At the last follow-up, the patient did not have retinopathy, peripheral neuropathy, symptomatic stroke, ischemic heart disease, or peripheral arterial disease.

## Discussion

**Figure 3.**
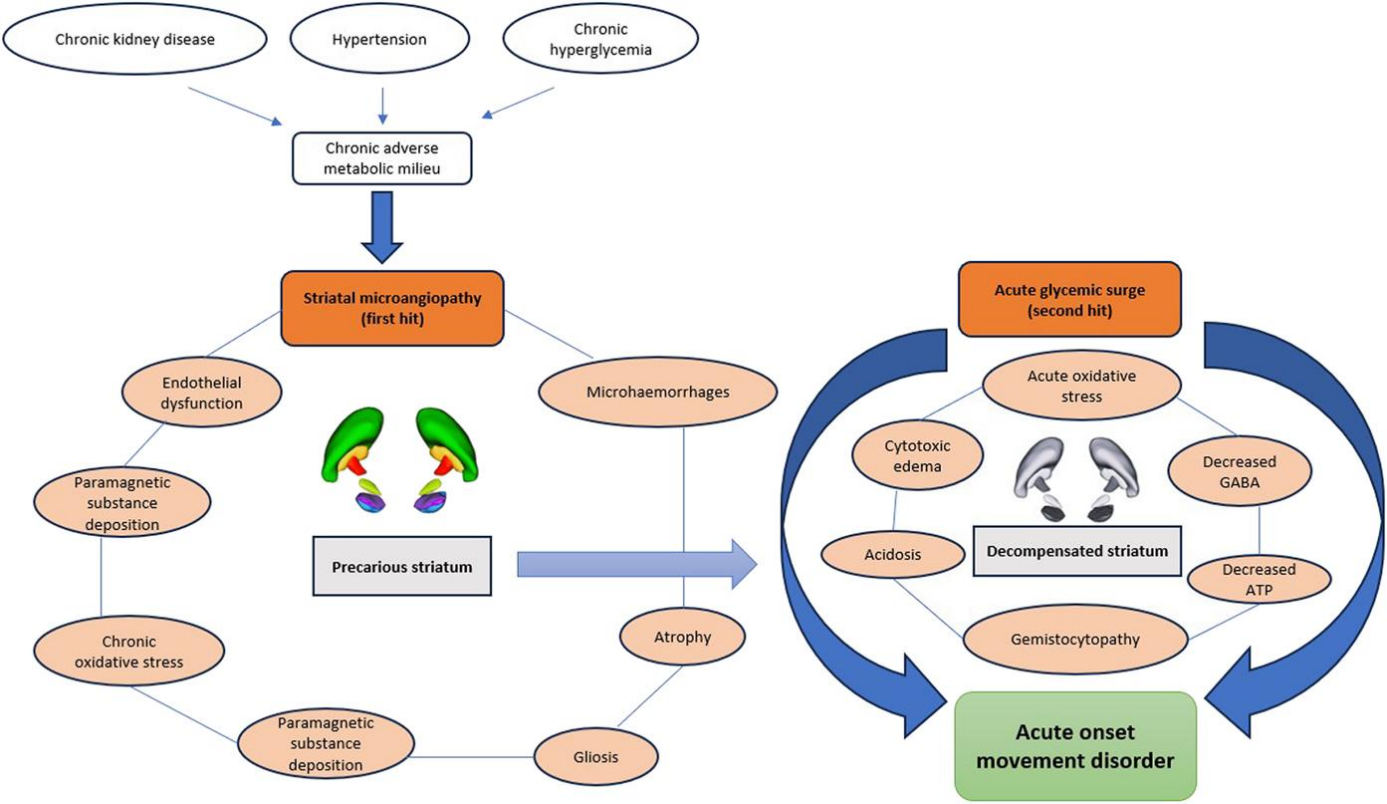
The “double-hit hypothesis” in the pathophysiology of diabetic striatopathy. Chronic hyperglycemia, hypertension, and chronic kidney disease lead to striatal microangiopathy, which is the “first hit” (acting in conjunction with other factors like endothelial dysfunction, microhemorrhages, paramagnetic substance deposition, atrophy, etc.) to produce a precarious striatum. A surge of hyperglycemia (“second hit”) leads to altered neurometabolism (depletion of γ-aminobutyric acid and adenosine triphosphate, acidosis, etc.) and development of gemistocytes and cytotoxic edema. These acute events ultimately decompensate the striatal activity, leading to the genesis of hyperkinetic movements.

While DS is typically associated with chronic, poorly controlled diabetes, it has frequently been reported as the first presentation of diabetes [8]. The pathophysiology likely involves metabolic derangement and regional vulnerability of the striatum, resulting in reversible cytotoxic edema or irreversible ischemic injury [2]. This case illustrates an uncommon presentation of DS secondary to corticosteroid-induced diabetes in a patient with CKD. High-dose glucocorticoids caused a glycemic surge in a previously euglycemic individual, triggering acute-onset movement disorder. Here we introduce the concept of the “double-hit hypothesis” of the pathophysiology of DS where underlying precarious striatum (first hit from microangiopathy due to hypertension, chronic hyperglycemia, CKD, etc.) is further jeopardized by the acute neurometabolic dysfunction due to an acute hyperglycemic surge (second hit), resulting in hyperkinetic movement disorder (Fig. 3).

In the present case, early recognition and aggressive glycemic control led to significant neurological improvement. However, residual chorea and gliosis on follow-up imaging suggested partial structural damage despite metabolic correction. A similar case has been described by Lee et al, where an octagenarian woman presented with chorea 2 months [blood glucose 440 mg/dL (24.44 mmol/L), HbA1c 12.0% (108 mmol/mol)] after starting dexamethasone (2 mg/day) for arthralgia [6]. The patient was managed with insulin therapy, and chorea subsided within 48 hours. Interestingly, after stopping the corticosteroid, by the next 3 months, the glycemic status reverted back to normal without requiring any antidiabetic drugs. As in our patient, follow-up after 1 year in the patient described by Lee et al had persistent subtle chorea [6]. However, unlike ours, the study did not document a follow-up brain MRI to document permanent structural damage. Chinthapalli et al described a case of DS in an octogenarian male who presented with hemiballismus with classic neuroradiological features of DS while going into hyperglycemic crisis induced by prednisolone used for polymyalgia rheumatica [7]. However, there was no definite documentation that his diabetes was truly secondary to corticosteroids alone.

Unlike the 2 cases described by Lee et al [6] and Chinthapalli et al [7], our patient had impaired renal status. Nephropathy, particularly with eGFR <60 mL/min/1.73m^2^, has been documented as a risk factor for the development of DS [9]. The underlying nephropathy possibly decreases the threshold of hyperglycemia-induced dysfunction of the striatum, which is particularly susceptible for metabolic insult. Uremic toxins, heavy metals (like aluminum, manganese, zinc, and calcium), guanidines, asymmetric dimethylarginine, endothelial dysfunction, smooth muscle dysfunction leading to autoregulatory failure, metabolic acidosis, hyperparathyroidism, etc. have been implicated in the pathogenesis of striatal dysfunction associated with nephropathy [9-11].

Although there is no definite cut-off of serum urea level in which clinical manifestation suggesting basal ganglia dysfunction occurs, it usually occurs in end-stage renal disease and particularly among patients with diabetes dependent on dialysis [10-12]. In this context, movement disorders resulting from bilateral basal ganglia lesions among end-stage renal disease patients are increasingly being recognized. These patients usually present with parkinsonian features, although chorea, ballism, choreoballism, and choreodystonia have also been described [13]. As these uremic patients frequently harbor diabetes, sometimes it becomes difficult to tell whether the movement disorder is chiefly attributable to dysglycemia or uremia. Imaging in uremia-induced striatal dysfunction usually reveals bilateral symmetrical involvement of the basal ganglia, hypodensity on computed tomography scan, hypointensity on T1, and hyperintensity on T2, and fluid attenuation inversion recovery sequences of MRI [12-14]. On the contrary, DS patients usually present with unilateral affliction of the basal ganglia with computed tomography showing striatal hyperdensity without mass effect and T1-MRI harboring hyperintensity (as in our patient) with variable intensity on T2 [15].

Our case illustrates a sobering clinical lesson. While steroid-induced diabetes proved reversible, the resulting DS left an indelible mark, much like a vanquished adversary that inflicts lasting damage before its demise. In high-risk individuals, such as those with CKD, corticosteroid therapy can precipitate profound metabolic complications leading to acute neurological complications. Early diagnosis and glycemic optimization are essential to ensure neurological recovery and prevent permanent sequelae.

## Learning Points

- Although classically linked with chronic poorly controlled type 2 diabetes, acute hyperglycemia from corticosteroid therapy may trigger DS even in previously euglycemic individuals.
- Impaired renal function may act as a facilitator in the development of DS by compounding striatal vulnerability to hyperglycemia via microangiopathy, endothelial dysfunction, and uremic toxins.
- Unilateral striatal T1 hyperintensity on MRI, in the absence of diffusion restriction, is characteristic of DS and helps differentiate it from uremic striatal dysfunction (which typically shows bilateral basal ganglia involvement).
- Early glycemic optimization may lead to substantial symptomatic recovery, though residual chorea and structural damage may persist.
- While hyperglycemia often resolves after tapering corticosteroids, the neurological insult can leave lasting deficits, underscoring the importance of its cautious use in high-risk patients such as those with CKD.

## Data Availability

All data related to this publication are available on request to the corresponding author.
